# Magic Blue Light: A Versatile Mediator of Plant Elongation

**DOI:** 10.3390/plants13010115

**Published:** 2023-12-31

**Authors:** Yun Kong, Youbin Zheng

**Affiliations:** School of Environmental Sciences, University of Guelph, 50 Stone Road East, Guelph, ON N1G 2W1, Canada; yunkong@uoguelph.ca

**Keywords:** blue LED, plant elongation, mechanisms, applications, future directions

## Abstract

Blue light plays an important role in regulating plant elongation. However, due to the limitations of older lighting technologies, the responses of plants to pure blue light have not been fully studied, and some of our understandings of the functions of blue light in the literature need to be revisited. This review consolidates and analyzes the diverse findings from previous studies on blue-light-mediated plant elongation. By synthesizing the contrasting results, we uncover the underlying mechanisms and explanations proposed in recent research. Moreover, we delve into the exploration of blue light-emitting diodes (LEDs) as a tool for manipulating plant elongation in controlled-environment plant production, highlighting the latest advancements in this area. Finally, we acknowledge the challenges faced and outline future directions for research in this promising field. This review provides valuable insights into the pivotal role of blue light in plant growth and offers a foundation for further investigations to optimize plant elongation using blue light technology.

## 1. Introduction

The elongation of plant stems represents a crucial growth trait in horticultural plant production, owing to its potential impact on plant development and yield. For example, the augmentation of stem elongation has been shown to confer benefits regarding the harvesting of microgreens and grafting of rootstocks. Conversely, the suppression of stem elongation can produce compact bedding plants and dwarf vegetable transplants, thereby enhancing their market value. Therefore, mediating plant elongation is one of the important goals for horticultural production. It is worth noting that chemical agents previously employed to stimulate or impede stem elongation are now facing increasing restrictions due to environmental concerns.

Light manipulation techniques have emerged as a sustainable method for altering plants’ elongation and, thus, morphology in controlled-environment production [1,2]. In contrast to open-field production, it is easier to manipulate the light environment in controlled-environment production. For example, in greenhouses and other types of indoor farms, plant elongation can be mediated through electrical lighting to modify the light environment, including light intensity, photoperiod, and spectral quality.

Among all light wavelengths affecting plant growth, blue light (BL; 400–500 nm) not only contributes to the normal function of plant photosynthesis but also plays an important role in regulating plants’ development and morphology. In many plants, light-mediated elongation can be influenced by BL, in addition to the ratio of red/far-red (R/FR) light. Sometimes, BL signaling dominates FR signaling in the mediation of plant elongation growth [3]. It has also been revealed that BL mediates stem elongation primarily through cryptochromes, while R light and FR light do so through phytochromes, which can also sense other wavelengths, including BL [1,4]. However, in contrast to earlier studies, diverse and even contrasting plant elongation responses to BL have increasingly been reported in studies using new lighting technologies such as LEDs. This review summarizes the different results from these studies (also including a series of relevant studies in our lab), explains the possible mechanisms involved, overviews the applications of blue LEDs to mediate plant elongation, and proposes future research directions.

## 2. A Scientific Consensus Has Been Revised by Discoveries from LED Lighting Studies

### 2.1. Blue Light Causes Plant Compactness: A Scientific Consensus

It is a prevailing scientific belief that BL generally causes plant compactness [5,6]. In a modified light environment where BL is filtered out from sunlight, plants demonstrate elongated stems, indicating the contribution of BL to light-inhibited plant elongation [7]. Also, a greater hypocotyl elongation of lettuce, spinach, and mustard has been observed in plants grown under high-pressure sodium (HPS) lighting than under metal-halide (MH) lighting, which emits a higher ratio of BL than HPS [8] (Supplementary Table S1), suggesting that blue-enriched light can inhibit plant elongation.

While both R light and BL affect stem elongation, it appears that BL has a greater inhibitory effect on plant elongation than R light. In certain plant species, such as soybeans, stem elongation cannot be effectively suppressed without adding BL to HPS lighting, whose light spectrum has a high R/FR ratio and a low BL proportion [9,10]. Also, early studies using broad-spectrum lighting sources have consistently shown that BL is more effective than R light in suppressing shoot/leaf elongation in a range of plant species [10,11,12,13,14,15]. 

The above beliefs and opinions have resulted from research that had BL in the background of either solar light or broad-band electrical lighting. Even for ‘monochromatic’ BL in the early studies, it was from a broad-band lighting source such as a blue-colored fluorescent lamp, which may have contained low levels of other spectral bands [16]. In this case, it is almost impossible to study plant elongation responses to BL wavelengths alone, due to the difficulty in isolating pure BL from these broad-band lights. 

### 2.2. Blue LEDs Alone Can Promote Plant Elongation

Unlike previous broad-band light sources, the utilization of LEDs, which emit narrow-waveband light, presents an opportunity to reassess the effects of pure BL on plant growth and development, as well as its interaction with other wavelengths. Also, LED studies have led to results contrasting with the prevailing scientific beliefs. For example, during our preliminary trials on ornamental plants (petunias, calibrachoa, geraniums, and marigolds), we observed a peculiar phenomenon whereby increasing the BL percentage in the lighting with a combination of blue and red LEDs (RB-LEDs) up to 100% (i.e., blue LEDs only) from 0% (i.e., red LEDs only) did not result in more compact plant growth, but instead led to greater stem elongation. In other words, compared with RB-LEDs (0% < B < 100%) and red LEDs, blue LEDs promoted plant elongation. Interestingly, other research groups have reported similar results regarding LED lighting in one or two of the same species we tested [17,18,19,20,21,22], although they have not conducted further investigations to elucidate this observation, except for a mechanism study on hormones by Fukuda’s group [17]. The findings of our lab, along with those of other researchers, have revised the prevailing scientific belief that BL causes plant compactness and has a greater inhibitory effect on plant elongation than red light.

To learn whether the promotional plant elongation under BL relative to R light is a common phenomenon, we consulted the relevant literature on LED studies, as listed in Table 1, Table 2 and Table 3. We found that there are reported discrepancies in the morphological responses to blue vs. red LED light for plants under conditions other than an *in vitro* environment. Specifically, blue LED light was found to promote stem or leaf elongation in eggplants [23,24], cherry tomatoes [25], cucumber [26,27], watermelon [28], sesame [29], arugula [30,31,32,33,34,35,36], kale [30,32,33,34], cabbage [30,32,33,34,37], sunflowers [16,38], peas [39], calibrachoa [19,40,41,42,43], petunias [17,21,22,40,41,42,43,44], marigolds [18,40,42,43], geraniums [40,42,43], tulips [45], and wild *Arabidopsis* [46,47,48], compared to red LED light (Table 1). On the other hand, blue LED light was observed to inhibit stem or leaf elongation in lettuce [24,49,50,51,52,53], cherry tomatoes [54], tomatoes [26,52,53,55,56,57,58,59], cucumber [52,53], radishes [52,53], pepper [60,61], bitter gourd [62], kale [63], mustard [31], impatiens [21,57,58], salvia [57,58], zinnia [21], chrysanthemum [64], spruce [65], rice [66,67,68], artichoke [69], mulberry [70], kiwi [71], coriander [72], bamboo [73], soybeans [52,53], maize [74], barley [75], cannabis [76], and wild *Arabidopsis* [77], compared to red LED light (Table 2). In addition, blue LED light was also found to have similar effects to red LED light on plant elongation in lettuce [78], pepper [52,53], mustard [30,32,33,34,36,79], kale [79], tomatoes [80,81], geraniums [16,41], marigolds [41], kalanchoe [64], poinsettias [64], and wheat [52,53] (Table 3).

It appears that under LED lighting, BL does not necessarily cause compact plants and can even result in stretching of plants relative to R light. Comparison of studies on plants’ elongation responses to blue vs. red LED light is complicated by the different plant genotypes, growth stages, LED lighting features, and cultivation conditions during the different trials (Table 1, Table 2 and Table 3). Even in the same trial, different durations of lighting treatment can also lead to varying plant elongation responses to blue vs. red LED light [88,92,93]. 

## 3. The Purity of Blue Light may Affect Plants’ Elongation Responses to This Light Wavelength

In light of the growing body of research on BL-promoted plant elongation from LED lighting, it is plausible to speculate that the absence of such effects in prior studies employing non-LED BL sources might be attributable to the presence of low levels of other light wavelengths, such as a high ratio of R/FR light, that activate phytochromes, thereby making the BL exhibit more suppressive effect on elongation growth than R light [43]. For instance, the blue-colored fluorescent lamp, which was previously one of the commonly used BL sources, was reported to have a R/FR ratio of 1.87 [11]. The white fluorescent lamp filtered through blue acetates, another previously utilized BL source, did not contain >700 nm light due to the filters employed [96]. In contrast to BL from non-LED lighting, blue LED light exhibits a much lower phytochrome photostationary state (PPS, an indicator of phytochrome activity), estimated as 0.5, compared to 0.9 for red LED light, as per the method established by Sager et al. [97]. Although the threshold value of PPS required to elicit an active phytochrome response remains a matter of debate, it is generally agreed that a PPS < 0.6 may provoke an inactive response [98]. The lower phytochrome activity in plants could potentially account for the elongated plants observed under blue LED lighting. Thus, we postulate that the effects of BL on plant elongation may, in some cases, be linked to phytochrome activity, which may differ under pure and impure BL sources. 

### 3.1. Adding Low-Level Red Light to Pure Blue Light Can Inhibit Plant Elongation, but This Can Be Reversed by Further Adding FR Light

**Figure 1:**
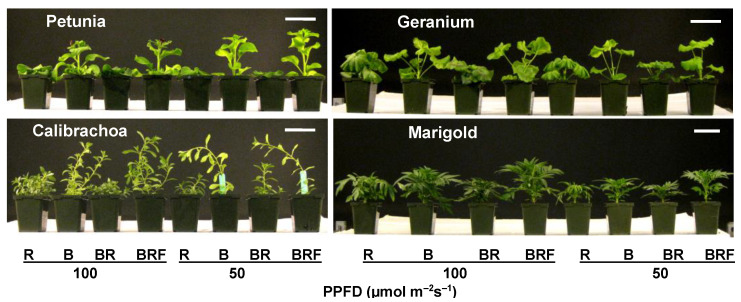
Plant elongation responses to pure and impure blue light in four ornamental plant species [43]. R = narrow-band red LED as a control treatment; B = pure blue light from a narrow-band blue LED; BR = impure blue light created by mixing B with a small amount (10% total PPFD) of R; BRF = impure blue light created by mixing BR with a small amount of far-red light, with red/far-red ≈ 1. The PPFD of the LED lighting was either 50 or 100 µmol m^−2^ s^−1^ for all treatments. The reference bar length in these pictures is 8.5 cm.

To examine the hypothesis proposed above, we conducted the first experiment involving four species of bedding plants: petunias, calibrachoa, geraniums, and marigolds [43]. In this experiment, in addition to pure BL from a blue LED (B; PPS = 0.5), we created a high-PPS impure BL (BR; PPS = 0.7) by adding a low level (10%) of R light to B, along with a low-PPS impure BL (BRF; PPS = 0.6) by further adding a small amount of FR light, with the R/FR ratio approximately equal to 1. After 14–20 days of lighting treatment, the pure BL (B) promoted plant elongation compared to R light (Figure 1) [43]. However, the high-PPS impure BL (BR) had the opposite effect and inhibited elongation growth to a similar or greater extent than pure R light. The low-PPS impure BL (BRF) restored the promotional effect observed with pure BL. The R/FR reversibility and the PPS changes suggest that the promotional effect observed with pure BL is linked to low phytochrome activity [43]. Under certain conditions, pure BL may need to co-act with R light to inhibit elongation growth by increasing phytochrome activity. Even for some species showing shorter plants under B vs. R LEDs (e.g., tomatoes), a combination of B with R LEDs (RB-LEDs) can inhibit plant elongation to a greater degree compared with B LEDs [26]. 

In our first experiment, a proportion of 10% R may not qualify as a low-level proportion for the high-PPS impure BL (BR). However, proportions of R lower than 6% in BR cannot be achieved by adjusting the LED lighting. An alternative approach involves adding gradually increasing amounts of FR light (i.e., gradually decreasing R/FR ratios) to BR, from 0 to 6%. In further experiments with bedding plants and microgreens, four impure BL treatments were established in addition to R and B [33,41,42]. These four impure BL treatments, denoted as BRF0, BRF2, BRF4, and BRF6, were created by blending B with a low-level (6%) R and further adding 0, 2, 4, and 6 µmol m^−2^ s^−1^ of FR light, respectively. It was found that BRF0 (PPS = 0.69) inhibited plant elongation compared with B, but the inhibitory effect reduced (or the promotional effect increased) gradually with the further addition of an increased level of FR. This was accompanied by decreasing PPS values from 0.69 (BRF0) to 0.65 (BRF2), 0.63 (BRF4), and 0.60 (BRF6). However, B did not show a greater promotional effect on plant elongation than BRF6, despite a lower PPS value (0.49 vs. 0.60). It appears that the plant elongation promoted by BL gradually became saturated once the PPS values decreased below 0.60 [33,41,42]. It is possible that the deactivated phytochrome contributes to the maximum elongation promotion by BL [33].

### 3.2. Adding Low-Level Wavelengths Other Than Red Light to Pure Blue Light Has Little Effect on Plant Elongation

Adding low-level R light to pure BL has a similar or greater inhibitory effect than that of R light on plant elongation, so it is also interesting to know how plant elongation responds to adding low-level ultraviolet-A (UVA) or FR light to pure BL. BL from some non-LED lighting sources has also been found to contain small amounts of UVA or FR light [99]. Considering this point, in a study on microgreens, we created an impure BL containing low-level UVA (BUA) by mixing B with a low level (7.5% of total PPFD) of UVA, along with another impure BL containing low-level FR (BF) by mixing B with low-level (10% of total PPFD) FR light [32]. BUA vs. B slightly inhibited elongation growth for some species at a PPFD of 100 µmol m^−2^ s^−1^, but BUA vs. R did not, except for mustard at 50 µmol m^−2^ s^−1^. BF vs. B slightly increased the hypocotyl length for arugula and mustard, as well as the petiole length for arugula. When considering all plant traits together, the effects of BUA and BF were similar to those of B, but different from those of R [32]. These findings imply that, relative to R light, the included low-level UVA or FR light plays a less important role in the inhibitory effect of impure BL on plant elongation. 

In addition to R, FR, and UVA light, blue-colored fluorescent lamps also contain low-level green (G) light (approximately 6% PPFD) and very low-level ultraviolet-B (UVB) light (<1 µmol m^−2^ s^−1^) [12,99]. Therefore, it is essential to investigate how impure BL containing only low-level UVB or green light affects plant elongation growth compared to pure BL and R light. To address this, we implemented one impure BL treatment (BUB) by mixing B with a low level of UVB and another impure BL treatment (BG) by mixing B with a low level of G light in a study on microgreens [34]. For arugula and kale, the elongation growth was slightly inhibited under BUB compared to B, whereas it was slightly promoted under BG for all species except arugula. Considering all plant traits together, the effects of BUB or BG were similar to those of B, but different from those of R [34]. Thus, relative to R light, the included low-level UVB or G light plays a less important role in the inhibitory effect of impure BL on plant elongation.

It appears that among the wavelengths possibly contained in impure BL, low-level R light has the greatest contribution to the inhibitory effect of impure BL on plant elongation. In the above studies, the calculated PPS values of BUB, BUA, BG, and BF were similarly low (<0.6) to those of B, but they were much less than those of BR, indicating that phytochrome activity plays an important role in plant elongation mediated by BL [32,34]. The contribution of phytochrome was also supported by a recent study on tomato plants’ response to the co-action of BL and G light. It was found that adding G light (20% of total PPFD) to pure BL significantly decreased the stem length, whereas G light hardly affected the stem length when added to a sole R or R+B mixture background [81]. Also, the *cry* mutants indicated that the reduction in elongation achieved by partially replacing BL with G light is independent of cryptochromes. Adding 20% G light to pure BL increased the PPS value from 0.51 to 0.58, but adding G light to R or RB had little effect on the PPS values, implying an involvement of phytochromes in this process [81]. 

## 4. Factors Affecting Plants’ Elongation Response to Pure Blue Light Relative to Red Light

As mentioned before, inconsistent results on plants’ elongation response to BL relative to R light have also been obtained from LED lighting studies. This may be related to differences in lighting features, plant factors, and cultivation conditions between different trials. However, each of the affecting factors needs to be tested in the same trials.

### 4.1. Lighting Features

Light intensity can interact with light quality to affect plant elongation. In the initial study, the impact of BL on plant elongation was tested using two PPFD levels of 100 and 50 µmol m^−2^ s^−1^. Recent LED-based research has reported that pure BL and R light at PPFD levels of 200 or 500 µmol m^−2^ s^−1^ inhibited elongation growth in some crops, but not all [52,53]. Furthermore, at PPFD levels of 30–50 µmol m^−2^ s^−1^, it has been shown that pure BL consistently inhibits elongation growth compared to R light in tissue-cultured plantlets for a broad range of species, including chrysanthemum [84], strawberry [95], grape [87,88], banana [82], *Cymbidium* [85], and *Doritaenopsis* [86]. To further examine the impact of pure BL relative to R light on plant elongation at a broader light intensity, our lab conducted a study on arugula and mustard seedlings under blue or red LED lighting, at PPFD levels ranging from 20 to 650 µmol m^−2^ s^−1^ [35]. It was found that the hypocotyl elongation of arugula was promoted by BL at all tested PPFD levels compared to R light, while for mustard the promotional effect was limited to higher PPFD levels, i.e., 250–650 µmol m^−2^ s^−1^. Additionally, for arugula, the promotion of hypocotyl elongation by BL decreased as the PPFD level increased [35]. The interaction effect of light intensity on BL-mediated plant elongation has also been found in other plant species [32,43].

The photoperiod can also interact with light quality to affect plant elongation. The majority of our BL-related studies employed a 24 h photoperiod, due to the consideration that plants’ elongation during dark periods is typically faster and may be influenced to a greater extent by subtle temperature differences between light treatments, as well as by trace light pollution (e.g., R or FR light) [43]. Although the use of continuous lighting (i.e., no dark period) can eliminate any confounding effects of light/dark switching on the elongation growth response to BL relative to R light, it may disrupt plants’ growth rhythms and could result in artifacts. To determine whether periodic lighting can influence the effects of BL on plants’ elongation relative to R light, the seedlings of arugula, cabbage, mustard, and kale were subjected to B or R LED lighting at a photoperiod of 24 or 16 h d^−1^ [30]. Regardless of the photoperiod, B consistently promoted elongation growth compared to R for arugula, cabbage, and kale. The promotional effects of BL on elongation were often more pronounced under 24 h vs. 16 h lighting. In a further study, with a photoperiod of 12 h d^−1^, B vs. R LED light also promoted plant elongation for arugula [31]. These findings suggest that the promotion of elongation growth by BL is not solely dependent on the 24 h lighting cycle, despite varying promotional magnitudes under different photoperiods.

BL with different peak wavelengths may have different effects on plant elongation. The PPS is very dynamic across the entire BL waveband, ranging from 0.41 to 0.60 [97]. A study using B LEDs with peak wavelengths ranging from 432 nm to 466 nm indicated that green perilla (*Perilla frutescens*) plant elongation increased as the peak wavelength decreased below 446 nm [100]. Also, when B LEDs with different peak wavelengths were used as supplemental lighting for producing two baby greens (Chinese kale and pak choi), the plants were taller under B-430 than under B-400 for both species, and also than under B-465 for Chinese kale [101]. In most of our studies, plants were examined under BL within a narrow range of peak wavelengths (440–455 nm). It is necessary to know whether the BL-mediated plant elongation in our tested species also differs across different peak wavelengths of BL and how its effects vary when compare with those of other wavelengths in addition to R light. To address these gaps in knowledge, the growth and morphology traits of mustard and arugula seedlings were investigated under BL with three different peak wavelengths (B1: 404 nm, B2: 440 nm, or B3: 455 nm), UVA light (385 nm), FR light (730 nm), R light (665 nm), and darkness [36]. It was found that B1, B2, and B3 had similar effects on hypocotyl elongation for both species, and the three BLs, compared to R, promoted plant elongation for arugula, regardless of the peak wavelength. Among the tested lights, BL had the greatest promotional effect on plant elongation for both species, despite having a smaller promotional effect than darkness [36].

### 4.2. Plant Factors

**Figure 2:**
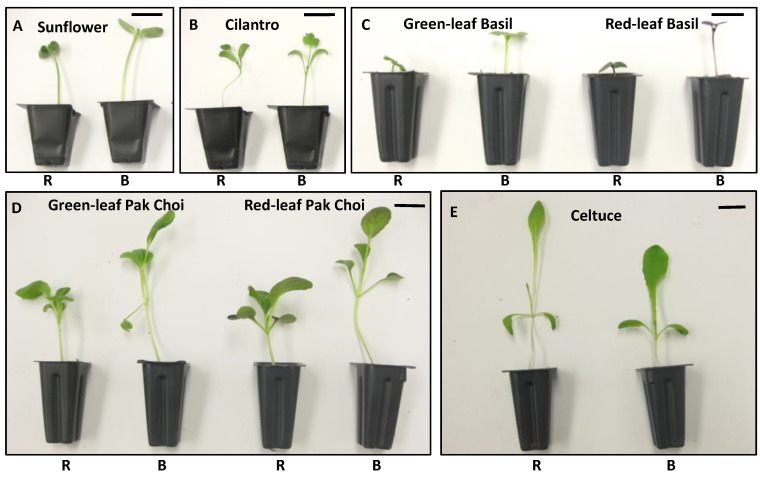
Plant elongation responses to blue or red LED light for different plant genotypes. R = red LED; B = blue LED. The PPFD of the LED lighting was 100 µmol m^−2^ s^−1^ for both treatments. The reference bar length in these pictures is 2.8 cm for (**A**,**B**) and 1.6 cm for (**C**–**E**). This figure is part of our unpublished works.

It has been found that BL-mediated elongation can vary between plant species and even cultivars [4,24]. In our initial experiments, only four ornamental species (petunias, calibrachoa, geraniums, and marigolds) were evaluated [40,41,42,43]. However, subsequent experiments expanded the scope to microgreens such as arugula, mustard, cabbage, and kale [30,31,32,33,34,35], and other microgreens/baby greens such as sunflower, cilantro, celtuce, basil, and pak choi (unpublished data; Figure 2), as well as the model plant *Arabidopsis* [46,47,48]. Most of the tested species exhibited increased plant elongation under BL relative to R light when exposed to continuous (24 h d^−1^) lighting at a PPFD of 100 µmol m^−2^ s^−1^, except for mustard, cilantro, and celtuce. For these three species, B LEDs still promoted elongation compared with RB-LED lighting but exhibited similar or inhibitory effects on plant elongation compared to R LEDs. In another experiment, for mustard, the promotional effect of B LEDs relative to R LEDs was observed under higher PPFD (>250 µmol m^−2^ s^−1^) rather than lower PPFD (<250 µmol m^−2^ s^−1^) [35]. Mustard has red pigmentation (anthocyanin) in its cotyledons, which could filter a part of R light and reduce its transmission to phytochromes. It is possible that as the light intensity increased, the transmitted R light level became high enough to induce an active phytochrome response, inhibiting elongation growth relative to BL. However, our recent trial comparing red- and green-leaf cultivars from the same species under BL and R light did not confirm this explanation (our unpublished data; Figure 2C,D). Even in red-leaf cultivars, B still promoted plant elongation compared to R, suggesting that pigment filtering may not entirely account for the species differences in BL response. Further study will be necessary to elucidate the varying plant elongation responses to B vs. R light for different plant genotypes.

In the initial experiment, the response of ornamental plant species to BL was assessed solely during the vegetative stage [102]. Subsequently, these same plant genotypes were examined at the transplant and flowering stages. During these growth stages, these plant genotypes exhibited similar promotional elongation responses to BL, except for marigolds and geraniums during the transplant stage, where the two species showed similar hypocotyl lengths under B vs. R LEDs [40,41,42]. However, in contrast to the ornamental plants, *Arabidopsis* exhibited distinct stem elongation responses to BL in seedlings versus mature plants. BL inhibited hypocotyl elongation in *Arabidopsis* seedlings but promoted main stem elongation in mature plants [47,48]. One possible explanation is that, in certain cases, phytochrome is not required for cryptochrome to inhibit hypocotyl elongation under BL [103]. Hypocotyl elongation only occurs in the early stages of plant growth, while main stem elongation continues until the later stages. Thus, it is possible that the involvement of phytochromes in BL-mediated elongation is less active during the early growth stage of *Arabidopsis* compared to the late stage. Another possible explanation is that cryptochrome activity is positively related to BL intensity [104], and that BL of the same intensity may trigger different responses in different plant organs due to different threshold values [105]. Therefore, it is possible that the cryptochrome activity under B at a light intensity of 100 µmol m^−2^ s^−1^ was high enough to inhibit hypocotyl elongation but not main stem elongation. However, unlike the *Arabidopsis* seedlings, species such as arugula, cabbage, and kale exhibited increased hypocotyl elongation under BL relative to R light [30,31,32,33,34,35]. It appears that whether or not the growth stage affects BL-mediated plant elongation varies with species.

### 4.3. Cultivation Conditions

Temperature variation can affect phytochrome activity and, thus, affect plant elongation under BL relative to R light. Studies on *Arabidopsis* indicated that phytochrome activity decreased with temperature increasing from 17 °C to 22 °C and 27 °C; accordingly, R light promoted hypocotyl extension at 27 °C, compared with 17 °C or 22 °C, but BL can repress high-temperature-mediated hypocotyl elongation through activated cryptochrome [106,107]. It has also been found that the maximum rosette growth rate of *Arabidopsis* under R light and BL is observed at 16 °C and 22 °C, respectively [108], implying different action temperatures between BL and R light. In our previous studies on B LEDs, only a constant temperature of around 23 °C was used. Through collaboration with the Texas A&M AgriLife Research Center, we investigated how temperature variations affected light-mediated plant elongation [31]. Arugula and mustard seedlings were grown indoors at 18 °C or 28 °C to compare plant elongation responses between R and B LED light. Regardless of temperature, B vs. R LED light promoted plant elongation in arugula, and the promotional effect was greater at 18 °C than at 28 °C, showing interactions between light and temperature. However, for mustard, there was no interaction between light and temperature with respect to plant elongation; plants were shorter under B vs. R light and were taller at 28 vs. 18 °C. In contrast to our previous studies, plant elongation decreased for mustard, and plant biomass decreased for both species under B vs. R light [31]. Possibly, a much shorter photoperiod (12 h d^−1^ vs. 16 or 24 h d^−1^) was used for this study, despite a similar PPFD (110 µmol m^−2^ s^−1^). The interactions of temperature and photoperiod with respect to BL-mediated plant elongation need further study.

Air humidity can modulate plants’ responses to BL, including plant elongation. Researchers from Norway found that when B LEDs were added to HPS lighting, tomato and cucumber plants under high relative humidity (RH; 90%) were taller compared with those under moderate RH (60%) [109]. They speculated that BL might have been used more efficiently for the development and function of chlorophyll and stomata under higher air humidity. In contrast, B LEDs inhibited shoot elongation for *in vitro* cuttings of *Rehmannia glutinosa* under no-ventilation conditions (at a higher air humidity), but they had a similar effect under ventilation conditions (at a lower air humidity), compared with R LEDs [92]. Despite the above studies, it is unknown how B LEDs as the sole lighting source affect plant elongation in seedlings and mature plants relative to R LEDs under different air humidity conditions.

**Figure 3:**
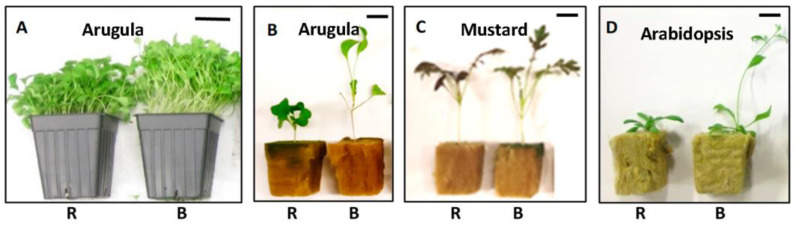
Plants’ elongation responses to blue or red LED light when growing at commercial planting intensity or in rockwool cubes. R = red LED; B = blue LED. The PPFD of the LED lighting was 100 µmol m^−2^ s^−1^ for both treatments. The reference bar length in these pictures is 4.3 cm for (**A**) and 2.5 cm for (**B**–**D**). This figure is part of our unpublished works.

Plants’ elongation response to BL relative to R light seems to be affected little by other cultivation factors, such as planting density and growth medium. In most of our studies on microgreens’ elongation response to B vs. R light, an evenly low planting density (with only one seedling per plug cell) was adopted to reduce the compound effect of plant–plant shading and provide the convenience to investigate the biometrics. However, in a follow-up experiment conducted on arugula and sunflowers at commercial (i.e., higher) planting densities, a similar promotional effect on plant elongation was observed under B vs. R LED light (unpublished data; Figure 3A). In most of our studies, a peat-lite mix was used for plant cultivation. However, for *Arabidopsis* [46,47,48] and some microgreens, such as arugula and mustard growing in rockwool cubes as an alternative medium, the plants also exhibited a similar elongation response to B vs. R LED light (unpublished data; Figure 3B–D).

## 5. Mechanisms Underlying Blue-LED-Promoted Plant Elongation

### 5.1. Shade-Avoidance Response

**Figure 4:**
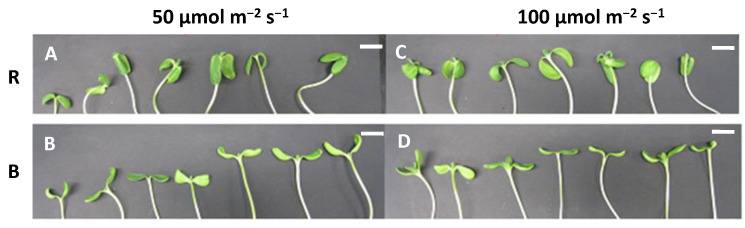
Leaf epinasty under red LEDs and leaf hyponasty under blue LEDs for sunflower microgreens. R = red LED; B = blue LED. The PPFD of LED lighting was 50 µmol m^−2^ s^−1^ (**A**,**B**) or 100 µmol m^−2^ s^−1^ (**C**,**D**) for both treatments. The reference bar length in these pictures is 2 cm. This figure is part of our unpublished works.

We have identified the promotion of stem elongation by blue LEDs as a shade-avoidance response (SAR), albeit with varying sensitivity across plant species [33,43]. In addition to elongated stems, other typical SARs have also been observed in other plant traits under blue LEDs. In mature plants, blue LEDs reduced the side-branching, chlorophyll content, leaf mass per unit area and/or increased individual leaf area, petiole length, biomass allocation to supporting structures, and/or advanced flowering time in petunias, calibrachoa, geraniums, and marigolds, compared with red LEDs [43]. Similar SARs were also observed in lettuce grown under narrow-band blue LEDs, which reduced the root dry mass, leaf chlorophyll content index, and leaf thickness compared with RB-LEDs [110]. In de-etiolated seedlings such as arugula, mustard, kale, and cabbage, blue LEDs resulted in longer petioles, smaller cotyledons, lighter plant color, or greater biomass allocation to hypocotyls [33]. Blue LEDs also caused leaf hyponasty in sunflower microgreens, which differed from the leaf epinasty under red LEDs (unpublished data; Figure 4), and the red-light-induced leaf epinasty in geraniums could be alleviated by blue LEDs [111]. Leaf hyponasty was also promoted in lettuce plants under blue vs. red LEDs, despite shorter stems [51]. Furthermore, the proteome changes in *Arabidopsis thaliana*’s response to blue LEDs relative to red LEDs also appear to be involved in the pathway of SARs [112].

**Figure 5:**
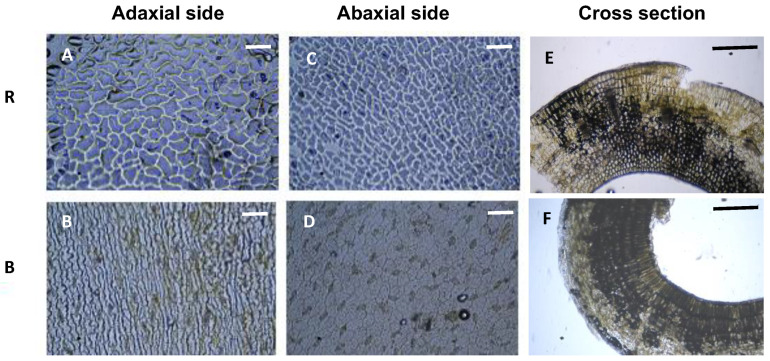
Epidermis cells of sunflower cotyledons under red or blue LED light. R = red LED; B = blue LED. The PPFD of LED lighting was 100 µmol m^−2^ s^−1^ for both treatments. The reference bar length in these pictures is 100 µm for (**A**–**D**) and 500 µm for (**E**,**F**). This figure is part of our unpublished works.

The BL-mediated SARs in morphological traits were partially supported by the changes in anatomical structure. In arugula, the hypocotyl epidermis demonstrated greater cell elongation under blue LEDs compared to red LEDs [34]. Similar results have been reported in *Arabidopsis* under low-level BL [113]. However, smaller cotyledon sizes in arugula, kale, and cabbage seedlings under blue vs. red LEDs resulted from decreased cell numbers rather than decreased cell size in the cotyledon epidermis; on the other hand, the leaf cell size increased to compensate for the reduced cell numbers [34]. Associated with decreased leaf thickness, some anatomical changes such as reduced palisades, and spongy tissue thickness were observed in lettuce leaves under blue LEDs compared with RB-LEDs [110]. The increased leaf hyponasty of sunflower seedlings under blue vs. red LEDs was due to the increased length of epidermis cells in the abaxial (or lower) sides of leaves (unpublished data; Figure 5), which also contributed to blue LED’s inhibition of leaf epinasty in geraniums under red light [111].

The SARs were primarily observed under BL with low PPS (such as B, BF, and BRF), but not under BL with high PPS (such as BR) [32,33,43]. Moreover, the BL-promoted SARs were more pronounced under lower light intensity [32,43]. In natural vegetative shade, plants experience both decreased ratios of R/FR and reduced intensity of BL, which can trigger plants’ elongation to compete for light as one of the SARs through reduced activity of PHY and CRY [114,115]. It is possible that the BL with low PPS is like an integrated shade signal that can be perceived by multiple photoreceptors such as PHY and CRY in plants. However, it remains unclear which factor—low BL level or low phytochrome activity—plays a more significant role in the BL-promoted SARs under specific conditions, necessitating further research.

### 5.2. Hormone Changes

GAs have been found to play an important role in BL-promoted plant elongation. In the case of petunias with elongated plants under blue vs. red LEDs, compared with other hormones, the contents of active gibberellic acid (GA) varied more markedly between blue and red light [116]. In stem tissues under blue LEDs, much higher levels of GAs (especially GA_4_ and GA_1_) were detected compared with those under red LEDs [17,116]. For the dwarf plants developed under red light, after the application of GA_3_, the plants’ height increased [17,116]. Under blue LEDs, the production of GA_20_-oxidase, one of the key enzymes in the synthesis of active GAs, might have increased in the shoot tips [117]. This has been supported by higher expression of *PhGA_20_ox-1S* and *PhGA_20_ox-2L*, two homologous genes for encoding GA_20_-oxidase in *Arabidopsis*, under blue LED treatment than under red LED treatment after 6 h of light treatment [22,116]. The increased enzyme production and gene expression were closely associated with higher contents of GAs under blue vs. red LEDs [117]. Another study in tomato seedlings also suggested that GA biosynthesis may be involved in the stem elongation of seedlings grown under low-BL conditions [118].

Auxin has been considered as a fundamental regulator of SARs induced by low R/ FR ratios [119]; however, it appears to play a minor role in BL-mediated plant elongation as an SAR. Low-R/FR-induced phytochrome inactivation stimulates auxin biosynthesis and induces hypocotyl elongation, petiole elongation, and leaf hyponasty in *Arabidopsis* [113]. Also, regulated transport of these enhanced auxin levels is essential to achieve elevated auxin concentrations in the hypocotyl to induce its elongation in *Arabidopsis* seedlings under low R/FR ratios [113]. For petunia plants, unlike GAs, the auxin content under blue LEDs was only slightly higher than that under red light treatment [116]. However, it is unclear whether other plant species have a similar response.

Brassinosteroids (BRs) have been shown to contribute to the SARs mediated by low-intensity BL. In *Arabidopsis*, the pathways for polar auxin transport, auxin biosynthesis, and gibberellin signaling that are involved in SARs under low R/FR ratios were not required for the SARs under low-intensity BL; in contrast, the BR response appeared to be required for the full expression of the SAS phenotype under low BL [120]. However, another study in *Arabidopsis* indicated that both auxin and BR play important roles in the regulation of enhanced hypocotyl elongation of seedlings in response to BL depletion, and only when both hormones are blocked simultaneously will the response be fully inhibited [113]. It is difficult to explain the contrasting results from the same species. Also, it is unknown how BR is involved in BL-promoted plant elongation as an SAR in horticultural crop species.

### 5.3. Involved Photoreceptors

We found that at least three photoreceptor systems are involved in the BL-promoted plant elongation as an SAR. Although phytochromes are primarily the receptors of R and FR light, the photoreceptors can also sense other wavelengths, including BL [1,121]. The blue LED has a low PPS below 0.6, which normally cannot induce an active phytochrome response [98]. Also, the R/FR reversibility, which is considered to be a hallmark of phytochrome action, indicates that the blue-LED-promoted elongation as an SAR is related to low-activity phytochromes [43]. Through further studies on *Arabidopsis* photoreceptor-deficient mutants and photoreceptor-overexpressing transgenic plants, we found that not only low-activity phytochromes but also low-activity cryptochrome 1 (CRY1), high-activity cryptochrome 2 (CRY2), and phototropins (including phot 1 and phot 2) are involved in the blue-LED-mediated responses [46,47,48]. Previous studies on *Arabidopsis* indicated that CRY1 plays a key role in BL-mediated inhibition of hypocotyl elongation, and that CRY1-mediated inhibition of hypocotyl elongation requires active phytochromes for full expression in some cases [104,122]. However, the detailed information about how CRY1 is deactivated under blue LEDs through crosstalk with other photoreceptors, such as phytochromes, is still unknown, especially for horticultural plant species.

One means of crosstalk between cryptochrome and phytochrome is the direct protein–protein interaction of the two photoreceptors, according to the studies of *Arabidopsis*. Phytochrome A (phyA) was previously found to directly interact with CRY1, and a direct interaction was also shown between phyB and CRY2, but these interactions were not demonstrated to be light-dependent [123,124]. Hughes et al. [125] reported a direct light-dependent interaction between phyB and CRY1, where CRY1 interacts specifically with the dark/FR state (Pr) of phyB, but not with the R light-activated (Pfr). Whether these interactions can explain the crosstalk between CRYs and PHYs to mediate plant elongation under blue LEDs is unknown.

Another means of crosstalk between cryptochrome and phytochrome is indirect interaction through common signaling molecules of these photoreceptors. For example, cryptochrome and phytochrome can both bind to the SUPPRESSOR OF PHYA-105 (SPA)/CONSTITUTIVE PHOTOMORPHOGENIC1 (COP1) complex to target certain sets of transcription factors for degradation [126]. They can also both bind to basic helix–loop–helix (bHLH) transcription factors, such as PHYTOCHROME INTERACTING FACTORs (PIFs) to control transcription [126]. In addition, it has recently been found that the blue-light inhibitors of cryptochromes (BICs) and photoregulatory protein kinases (PPKs) may also play roles in the cryptochrome-phytochrome coaction [127].

**Figure 6:**
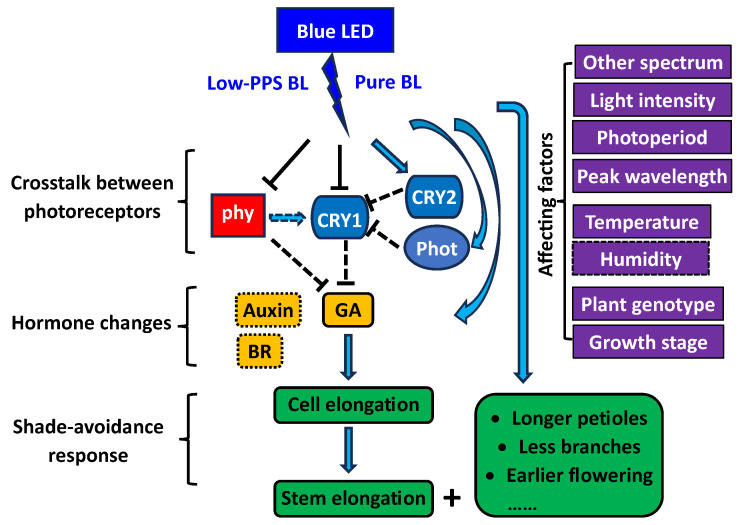
A proposed simple model for explaining the mechanisms involved in blue-LED-promoted plant elongation. BL = blue light; PPS = phytochrome photostationary state; phy = phytochrome; cry = cryptochrome; phot = phototropin; GA = gibberellic acid; BR = brassinosteroid. 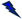 Light stimulus; 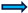 promotional signal; 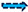 speculated promotional signal; 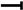 inhibitory signal; 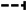 speculated inhibitory signal; 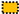 speculated involved hormone; 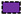 speculated affecting factor. The proposed model is based on the key findings from our previous studies [30,31,32,33,34,35,36,40,41,42,43,46,47,48], except for the GA signal from Fukuda’s group [17].

Based on the key findings of the relevant studies, especially the research conducted in our lab, we propose a simple model (Figure 6) to explain the underlying mechanisms involved in blue-LED-promoted plant elongation.

## 6. Application of Blue LEDs in Mediating Plant Elongation for Controlled-Environment Production

**Figure 7:**
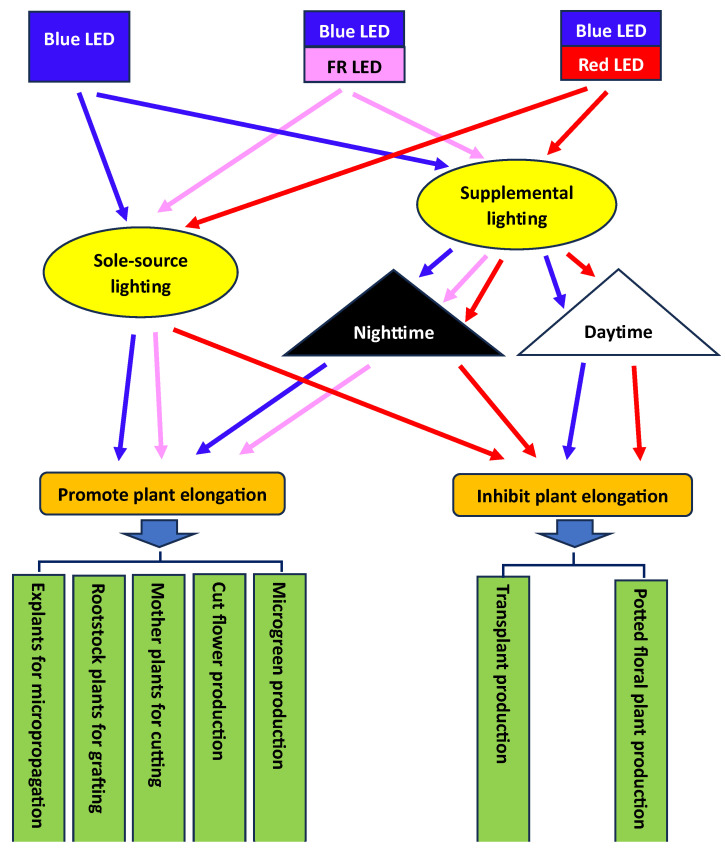
Potential ways to apply blue LEDs in plant production in a controlled environment. FR = far-red.

Considering that BL-mediated plant elongation can be affected by phytochrome activity, the application of blue LEDs, alone or in combination with R or FR LEDs, either as the sole source or as supplementary lighting, would potentially affect plant elongation differently during daytime or nighttime. Therefore, we propose different potential ways to apply blue LEDs in controlled-environment plant production for varying purposes (Figure 7). Most of these methods have been tested in relevant studies in our lab or by other groups.

### 6.1. Plant Propagation

#### 6.1.1. Promoting Explant Elongation for Micropropagation

Nodal and internodal explant culture stands as a straightforward and efficient technique for micropropagation. Nonetheless, certain plants, like *Paphiopedilum* and *Nepenthes*, present challenges due to their short and poorly defined internodes. Consequently, obtaining precisely delineated nodal and internodal explants for micropropagation becomes a formidable task. Moreover, the dense arrangement of leaves on these plants complicates the process of surface decontamination for explants [128]. 

For *P. delenatii*, one-month-old *ex vitro* single-node shoots (1.5–2.0 cm length) were grown under various light conditions, including blue or red LEDs alone, mixtures of blue and red LEDs, and darkness, for examinations of the shoot elongation. The best stem elongation was obtained under blue LEDs (100%B) after four months of culture [129].

#### 6.1.2. Promoting Hypocotyl Elongation of Rootstock Plants for Grafting

Producing seedlings with long hypocotyls is desirable in vegetable grafting. Longer hypocotyl lengths in the rootstock would both allow easier grafting and reduce the risk of scion exposure to the soil [130]. Although there have been many studies on the application of FR LEDs to promote hypocotyl elongation of rootstock for vegetable grafting [130,131,132], limited studies have reported the application of blue LEDs.

A short-term (10-day) pre-grafting lighting with blue LEDs at 15 µmol m^−2^ s^−1^ promoted plant elongation, increased the leaf number and size, and increased the graft–take ratio in tomato seedlings compared with darkness [133]. However, its beneficial effects were less than those of white fluorescent light or natural light. 

#### 6.1.3. Mediating Shoot Elongation of Mother Plants for Cuttings

*Campanula* mother plants have short shoots, and it is difficult to harvest cuttings; therefore, producing mother plants with long and thick side branches without flower buds is important for high-quality cuttings [134]. For indoor-grown *Campanula* mother plants, our lab has developed a three-stage lighting strategy, i.e., sequential lighting with red, blue, and RB-LEDs at three stages, aimed at increasing the number of side branches, promoting shoot elongation, and enhancing shoot thickness, respectively [134]. The dynamic lighting increased the side branch numbers and plant height without inducing flowering, meeting the target height (≈7.5 cm) for machine harvesting. Furthermore, the dynamic lighting improved the upright growth of side branches and did not affect the cutting quality or rooting. Overall, dynamic lighting with blue and red LED light has the potential to benefit the controlled-environment production of *Campanula* cuttings if the lighting strategy is further optimized. 

### 6.2. Transplant Production

For transplant production of vegetables or ornamental plants, normally the high-quality seedlings have compact canopies, developed root systems, high chlorophyll concentrations, and the ability to withstand transplanting shock [135,136]. Blue LEDs can be applied alone or in combination with other wavelengths as a sole or supplemental lighting source to mediate plant elongation as well as other quality indices during transplant production.

#### 6.2.1. Sole-Source Lighting with a Combination of Blue and Red LEDs Can Produce Compact Transplants under Indoor Conditions

Sole-source lighting with RB-LEDs is commonly used for indoor transplant production. A recent study in our lab indicated that RB-LEDs (15%B) can potentially replace fluorescent light, but the trichromatic lights appear to be unnecessary for the indoor production of compact gerbera transplants [137]. Compared with red or blue LEDs alone, RB-LEDs (50%B) caused more compact seedlings in bedding plants such as impatiens, petunias, and salvia [57]. In addition to more compact transplants, RB-LEDs (50%B) also promoted the post-transplanting growth of lettuce plants, due to higher biomass and antioxidant activities in the transplants, compared with red or blue LEDs alone [50]. Studies on cucumber and tomato seedlings have indicated that the lack of either blue or red light negatively affects early development, but BL appears to play a more critical role than red light [55,80,138]. 

Increasing the BL proportion in RB-LED lighting can not only promote plant compactness but also affect other plant traits. In cucumber seedlings, with the increase in the BL percentage in RB-LED lighting from 10% to 75%, the hypocotyl length, leaf area, and shoot biomass decreased, but the chlorophyll content increased, compared with red LEDs only [27]. In three bedding plants (impatiens, petunias, and salvia), when the BL percentage in RB-LED lighting increased from 6% to 50%, the plant height decreased by 23–50% and the leaf area decreased by 17–50%, while there was a decrease in shoot biomass for petunias and salvia and an increase in flower buds for impatiens, compared with red LEDs only [58]. 

The BL proportion in RB-LED lighting can be optimized based on multiple plant responses aside from plant compactness, but the optimal BL proportion seems to vary between plant species. Under sole-source lighting at a PPFD of 100 µmol m^−2^ s^−1^ and a photoperiod of 18 h, the optimal blue proportion in RB-LED lighting was 10% for cucumber seedlings and 30–50% for tomatoes [59]. Under RB-LED lighting at 300 µmol m^−2^ s^−1^ for 12 h d^−1^, the optimal BL percentage was 25% for sweet pepper and eggplant transplants, which showed the best performance not only in compact morphology but also in robust growth, with the highest seedling index value [23,60]. A similar optimal BL percentage has been identified in rice seedlings grown under RB-LED lighting at 100 µmol m^−2^ s^−1^ for 12 h d^−1^ [66].

The decision on the optimal proportion of BL in sole-source LED lighting needs to consider the specific goal(s) of the propagators. If plant compactness is the priority goal, as little as 6%B in RB-LED lighting at 160 µmol m^−2^ s^−1^ for 18 h d^−1^ can elicit compact transplants in bedding plants such as impatiens, petunias, and salvia [58]. For most plant species, at least 13%BL can be included in sole-source LED lighting to produce compact transplants [139]. In addition to controlling the stem length, the node position of the first flower truss is also crucial for the production of high-quality tomato seedlings in Japan [56]. Sole-source RB-LED lighting with less than 50%B and a BL intensity of 75 µmol m^−2^ s^−1^ has been recommended to suppress spindly growth and promote flowering during tomato seedling growth [56]. In commercial production, the decision of optimal BL proportion in LED lighting can also be related to economics, since BL requires more energy per photon [139].

#### 6.2.2. Supplemental Lighting with Blue LEDs Only or Their Combination with Red LEDs Can Produce Compact Transplants in Greenhouse Conditions

Blue LEDs alone can be used as a supplemental lighting (SL) source for the greenhouse production of compact transplants. In cucumbers, supplemental blue LEDs at 15 µmol m^−2^ s^−1^ with high-pressure sodium (HPS) lamps (90 µmol m^−2^ s^−1^) for 18 h d^−1^ not only decreased hypocotyls’ elongation, but also increased the leaf area, increased the fresh and dry weight, and enhanced their development, compared with HPS only [140]. In the same species grown in a greenhouse under low-intensity sunlight (about 2.7 mol m^−2^ d^−1^), 10 days of SL with blue LEDs relative to white, red, or green LEDs (at 120 µmol m^−2^ s^−1^ for 10 h d^−1^; 4.3 mol m^−2^ d^−1^) caused more compact plants with shorter stems and smaller leaf areas, despite similar shoot biomass [141]. Furthermore, after transferring to full sunlight (10.7 mol m^−2^ d^−1^), plants from the blue LED treatment developed similar leaf areas and 15% higher shoot biomass, showing better acclimation ability compared to other spectral treatments [141].

Blue LEDs in combination with red LEDs (RB-LEDs) can also be used as an SL source for the greenhouse production of compact transplants. For cucumbers and tomatoes, regardless of the natural light level (5–25 mol m^−2^ d^−1^), SL with RB-LEDs (4–16%B; PPFD = 54 µmol m^−2^ s^−1^; DLI = 3.6 mol m^−2^ d^−1^) resulted in compact transplants while improving transplant quality compared with no SL [142,143]. In six tomato cultivars grown in a greenhouse, SL with RB-LEDs (5–20%B; 61 µmol m^−2^ s^−1^; 5.1 mol m^−2^ d^−1^) reduced the hypocotyl elongation and increased the hypocotyl diameter, epicotyl length, shoot dry weight, leaf number, and leaf expansion relative to no SL under changing solar DLIs, from 0.4 to 19.1 mol m^−2^ d^−1^ [144]. In greenhouse-grown seedlings of bedding plants (including *Antirrhinum*, *Catharanthus*, *Celosia*, *Impatiens*, *Pelargonium*, *Petunia*, *Tagetes*, *Salvia*, and *Viola*), SL with RB-LEDs (15–30%B) at 100 µmol m^−2^ s^−1^ for 16 h daily reduced plant height by 9% to 55% and increased the stem diameter by 8% to 16%, showing a similar or higher transplant quality compared to HPS lamps [135].

For SL with RB-LEDs, the optimal BL proportion varies in different situations. In the greenhouse production of transplants, within the BL percentage range of 0–30%, 15%B in RB-LEDs was found to be optimal for bedding plants when used as SL [135]. However, for six cultivars of tomato transplants grown in a greenhouse under SL with RB-LEDs, the optimal BL proportion within the range of 0–20% varied between cultivars [144]. Also, in greenhouse-grown cucumber transplants, the seedling morphology was not different among RB-LEDs with different B%, especially under high natural light levels, and the plants did not even show a more beneficial response to RB-LEDs compared with red LEDs under 5–24 mol m^−2^ d^−1^ of solar DLI [142,143]. In this case, the impact of BL appears to be minimal, especially when background solar irradiance provides a sufficient amount of this wavelength [139].

### 6.3. Floral Plant Production

To meet the marketing requirements, not only are earlier flowering and more flowers beneficial to commercial growers, but also a compact plant morphology is helpful for production of potted floral plants, while an elongated stem is desired for production of cut flowers. Blue LEDs, alone or in combination with other LEDs, depending on the production purpose and plant genotype, can be used for mediating plant elongation as well as flowering in floral crop production. 

#### 6.3.1. Promoting Plant Compactness in Potted Floral Plant Production

The application of blue LEDs in combination with red LEDs (RB-LEDs) as sole-source lighting can produce compact potted floral plants. In roses, sole-source lighting with RB-LEDs (20%B) at 100 µmol m^−2^ s^−1^ for 20 h d^−1^ decreased the plant height, leaf area, and shoot biomass and increased the proportion of dry mass allocated to the leaves, without affecting flowering, compared to HPS lamps [145]. Indoor production with sole-source lighting with RB-LEDs (30%B) at 500 µmol m^−2^ s^−1^ for 16 h d^−1^ also led to more compact plants in four potted floral plants (primulas, marigolds, treasure flowers, and stock plants), while causing higher numbers of flower buds and fewer days to flowering compared with greenhouse production under natural light [91].

In addition to sole-source lighting, supplemental lighting (SL) with blue LEDs can also affect the compactness of potted floral plants. When narrow-band blue LEDs were used for daytime SL in the greenhouse, their effect appeared to be dependent on the natural background light level and the presence of FR light. In potted petunias, in late spring when the natural irradiance is higher (2.33 mol m^−2^ h^−1^), SL with blue LEDs at 50 µmol m^−2^ s^−1^ for 16 h d^−1^ in an FR-deficient environment inhibited stem elongation similarly to red LEDs, but in early spring when the natural irradiance was low (1.35 mol m^−2^ h^−1^), the SL with blue LEDs did not inhibit but, rather, promoted stem elongation and plant flowering compared to red LEDs [146].

Unlike blue LEDs alone, RB-LEDs can be more reliably used for daytime SL to produce compact potted floral plants, despite varying sensitivity among plant species. For potted poinsettias, strict control of plant height is essential in production, and RB-LEDs (20%B) at 100 μmol m^−2^ s^−1^ for 10 h d^−1^ were successfully used as an SL source in greenhouses or growth chambers to produce compact plants [147]. Compared with HPS lamps, the plants were 20–34% shorter and did not delay bract color formation, visible cyathia, or flowering, despite decreases in the leaf and bract area, chlorophyll content, and total dry matter accumulation [147]. Similarly, for potted geranium plants, supplemental RB-LED lighting with 45%B at 90 μmol m^−2^ s^−1^ for 16 h d^−1^ promoted canopy compactness, early flowering, and increased flower numbers compared with HPS [148]. However, species-specific responses have been reported for potted roses, chrysanthemums, and campanulas grown in a greenhouse. SL with RB-LEDs (40%B) at 200 μmol m^−2^ s^−1^ and 16 h d^−1^ reduced plant height while increasing the biomass in roses and chrysanthemums, but not in campanulas, compared with white or red LEDs [149]. 

#### 6.3.2. Promoting Plant Elongation in Cut Flower Production

In the winter production of chrysanthemums, a short-day (SD) plant, for cut flowers, electrical lighting is used to create long days (LDs) routinely for 2–3 weeks before the onset of short days to meet the required specific stem length, but this delays the transition to flowering. Research has shown that blue LEDs can be potentially used as a lighting source to extend the photoperiod during SD conditions for controlled-environment production of cut chrysanthemum flowers to promote stem elongation without inhibition of flowering. For example, a 4 h EOD treatment with blue LED light of 10 μmol m^−2^ s^−1^ after 9 h of daytime lighting with white LEDs at a PPFD of 180 µmol m^−2^ s^−1^ increased the plant height, leaf number, and leaf area without delaying the flowering time or reducing the flower number [150]. Also, EOD illumination with BL at 70 µmol m^−2^ s^−1^ for 4 h daily did not inhibit the flowering of chrysanthemums growing under 12 h daytime lighting with white fluorescent light at 70 µmol m^−2^ s^−1^ [151]. Furthermore, for plants growing under RB-LEDs (20%B) at 100 µmol m^−2^ s^−1^ for 11 h daily, a long-day treatment with 4 h EOD or 13 h overnight exposure to blue LEDs at 100 µmol m^−2^ s^−1^ did not inhibit flowering but did promote stem elongation [152,153]. 

It is worthwhile to note that different plant responses to prolonged-photoperiod lighting with blue LEDs have been found in chrysanthemums growing under different background light conditions as well as different reference lighting. Under an 11 h daytime condition, 4 h EOD SL with blue LEDs at 40 µmol m^−2^ s^−1^ inhibited chrysanthemums’ flowering in a greenhouse with daytime solar light, but not in a growth chamber with daytime lighting from RB-LEDs (40%B) at a PPFD of 100 µmol m^−2^ s^−1^, despite the increased stem length in both the greenhouse and chamber [154]. In contrast, for chrysanthemums growing indoors under sole-source lighting with fluorescent lamps at 150 µmol m^−2^ s^−1^ for 12 h, a 4 h nightly interruption with blue LEDs at 1.7 µmol m^−2^ s^−1^ reduced the daily internode elongation rate by about 60% compared with fluorescent lamps at 150 µmol m^−2^ s^−1^, and the inhibitory effect of blue LEDs was maintained not only in the nighttime interruption period but also in the subsequent dark and light periods [155].

In addition, blue LEDs can also be combined with other LEDs to mediate the plant morphology and flowering of chrysanthemums. In a greenhouse, 4 h of supplemental lighting with blue LEDs combined with FR LEDs (75%B + 25%FR) enhanced stem elongation and promoted early flowering [156]. In a walk-in growth chamber, nightly interruption with 2 h of blue LEDs first and then 2 h of FR LEDs at an intensity of 10 µmol m^−2^ s^−1^ promoted both plant elongation and the number of flowers per plant, compared with 10 h short-day treatments [157]. In a growth chamber under 13 h daytime lighting with white LEDs at a PPFD of 180 µmol m^−2^ s^−1^, 4 h of EOD lighting with blue LEDs at 10 µmol m^−2^ s^−1^ promoted flowering and increased plant height [150].

Blue LEDs have also been found to show a promotional effect on the elongation of some other cut flower species. In tulips, sole-source lighting with blue LEDs for 12 h d^−1^ at a PPFD of 200 µmol m^−2^ s^−1^ increased the cut flower stem length and cut flower fresh weight compared with red LEDs [45]. Sole-source lighting with blue LEDs for 16 h d^−1^ also caused taller tulip plants than red or white LEDs and resulted in an earlier sprouting and flowering and a higher biomass compared with darkness [158]. 

### 6.4. Microgreen Production

Microgreens are typically harvested at 7 to 21 days from seeding, and a minimum height of 5 cm is required before the final harvest [159]. In recent years, the trend in commercial microgreen production has been to switch from hand to machine harvesting to reduce labor costs. However, machine harvesting of microgreens with hypocotyls shorter than 5 cm can be challenging. LED lighting can be used to mediate the hypocotyl elongation of microgreens during controlled-environment production.

#### 6.4.1. Application of Blue LEDs in Daytime Lighting to Promote Hypocotyl Elongation

RB-LEDs have been popularly used as daytime lighting sources for indoor-grown microgreens, and the BL proportion in RB-LEDs can be optimized to promote plant elongation while maintaining the yield and other quality traits. For daytime sole-source lighting at 300 µmol m^−2^ s^−1^ for 16 h d^−1^ during indoor microgreen production, the BL proportion in RB-LEDs (between 5–30%B) was optimized in terms of plant elongation, yield, and other appearance qualities for cabbage at 15%B, and at 5%B for kale, arugula, and mustard [160]. 

Although the BL proportion in RB-LEDs can be optimized, it can still cause shorter plants than blue LEDs. For example, under RB-LED lighting at 220 µmol m^−2^ s^−1^ for 18 h d^−1^, RB-LEDs with only 10%B reduced the hypocotyl length in *Brassicaceae* microgreens compared to blue LEDs only [79]. However, microgreens grown under either blue or red LEDs alone cannot meet the commercial requirements in terms of both plant height and appearance quality, so a potential approach using sequential lighting, with blue LEDs first to increase plant height and then red LEDs to improve leaf size and plant color (i.e., temporal combination of blue and red LEDs), has been suggested by our lab to address this problem [30]. Another approach developed by us to address the problem of short plants under RB-LED lighting is delaying the start of the lighting for several days—in other words, using early-stage dark treatment [161,162], since darkness, relative to RB-LED lighting, can also promote hypocotyl elongation during the early development stage of plants [102]. However, this approach is better for larger-seed species such as sunflowers, due to a potential yield loss in smaller-seed species such as arugula, despite the promotion of plant elongation in both species [161]. 

#### 6.4.2. Application of Blue LEDs in Nighttime Lighting to Promote Hypocotyl Elongation

For indoor-grown microgreens under electrical lighting, blue LEDs can be used for nighttime lighting to promote microgreen elongation without affecting yield or quality. For two microgreen species grown indoors under sole-source lighting with RB-LEDs (20%B) at a PPFD of 300 µmol m^−2^ s^−1^ during 16 h of daylight, nighttime lighting with blue LEDs alone at 20 µmol m^−2^ s^−1^ for 8 h or at 40 µmol m^−2^ s^−1^ for 4 h increased plant height by 34% and 18% for mustard and arugula, respectively, compared with no nighttime lighting [163]. Nighttime lighting with 20 µmol m^−2^ s^−1^ blue LEDs and 20 µmol m^−2^ s^−1^ FR LEDs together for 8 h further improved the promotional effect on elongation. The 8 h lighting with blue LEDs alone also increased the fresh weight of arugula by 12% compared to darkness. Additionally, nighttime treatments with blue LEDs, alone or in combination with FR LEDs, increased the chlorophyll content index, leafy index, or dry matter content, depending on the species [163]. 

During winter greenhouse microgreen production, overnight lighting with low-level blue LEDs alone can also promote plant elongation while improving the appearance quality and crop yield, without negatively affecting nutritional quality. For mustard and arugula microgreens, overnight lighting with 14 µmol m^−2^ s^−1^ from blue LEDs promoted stem elongation by 16% and 10%, respectively, and increased crop yield by 32% and 29%, respectively, compared to no overnight lighting [164]. Furthermore, blue LEDs increased the cotyledon area in mustard and the leaf mass unit area in arugula, and they enhanced the cotyledon color in both species, without affecting the total chlorophyll, carotenoid, and phenolic contents. However, overnight lighting with 14 µmol m^−2^ s^−1^ from FR LEDs did not have a positive effect on the above plant traits compared to blue LEDs [164].

## 7. Future Research Directions

BL-mediated plant elongation involves intricate molecular, physiological, and ecological mechanisms. Further research is needed to explore the detailed molecular and physiological mechanisms underlying plant elongation in response to blue LEDs (or pure BL) through (1) identifying key signaling components, including receptors, kinases, and transcription factors, that are involved in BL-promoted elongation; and (2) investigating gene expression patterns, protein–protein interactions, and hormone signaling pathways. Also, it is necessary to study how plant elongation responds to the co-action of BL with other light wavelengths, both individually and collectively, through (1) utilizing advanced imaging techniques to understand spatiotemporal regulation and (2) integrating multi-omics data for a comprehensive understanding of the complex crosstalk between different signaling pathways.

For application research, while blue LEDs are already used for plant elongation, further research is required for the following: (1) Optimization of BL manipulation by considering plant-species-specific responses, lighting intensity, and lighting duration, while integrating BL manipulation with environmental factors in controlled environments through integrated environmental control systems for optimal plant growth. (2) Development of dynamic, alternate, intermittent lighting strategies with blue LEDs and other LEDs for specific plant species and growth stages. (3) Application of new light sources, such as laser light and plasma lighting, for manipulating BL, while exploring their potential in optimizing the peak wavelengths and proportions of BL for specific production purposes.

It is also worth noting that caution should be taken in conducting relevant research in the future. For example, in addition to lighting sources, light contamination from neighboring treatment zones would affect the BL purity and, thus, result in contrasting results. Also, the application of blue LEDs as nighttime lighting or daytime lighting in a greenhouse may cause contrasting morphology for some plant species, due to different background light conditions. Further challenges arise due to inconsistencies in light intensities among light-quality treatments, which may introduce confounding factors and yield inaccurate conclusions about the effects of light quality. Therefore, it is important to separate different lighting treatments to avoid neighboring light pollution and keep uniform environmental conditions across different light treatments, as well as within the same treatment, when setting up the lighting experiment.

## 8. Conclusions

Recent developments in LED technologies have led to the development of narrow-band electrical lighting for crop production in controlled environments. The discovery that narrow-band BL from LED lighting may promote plant elongation has challenged the current scientific consensus, which was established on the knowledge gained by using traditional broad-band BL sources. A series of studies have further confirmed this discovery and have explored the underlying mechanisms and practical applications. However, recent studies have reported varying and even contrasting elongation responses to blue LEDs among different plant genotypes, development stages, and environmental conditions. This has revealed how little is known about the physiology involved in BL-mediated plant elongation responses. Future studies based on this discovery will need a collaborative effort of researchers from different fields.

## Figures and Tables

**Table 1:** Plant elongation promoted by blue light relative to red light from sole-source LED lighting.

Plant Name	Genotype/Variety/Cultivar/Strain	Growth Stage	Elongation Growth Biometrics	Blue/Red LED Peak Wavelength (nm)	PPFD (µmol m^−2^ s^−1^)	Photoperiod (h d^−1^)	Air Temperature (℃)	RH (%)	Treatment Days	Reference
Arabidopsis (*Arabidopsis thaliana*)	Col-0, *phot1*, *phot2*	Mature plants	Stem length	455/660	100	24	23	65	20	[46]
	Col-0, *cry1*, *cry2*, *cry1cry2*, *CRY2-OX*	Mature plants	Stem length	455/660	100	24	23	65	18	[48]
	*cry1*, *cry1cry2*	Mature plants	Hypocotyl length	455/660	100	24	23	65	18	[48]
	col-0	Mature plants	Stem length	455/660	100	24	23	65	18	[47]
Arugula (*Brassica eruca*)	‘Rocket’	Seedlings	Hypocotyl length	450/660	50 or 100	24	23	50–55	13	[32]
	‘Rocket’	Seedlings	Hypocotyl length	455/660	100	24	23	65	8	[33]
	‘Rocket’	Seedlings	Hypocotyl length	440/665	100	24 or 16	22	70	8	[30]
	‘Rocket’	Seedlings	Hypocotyl length	455/660	100	24	22	70	8	[34]
	‘Rocket’	Seedlings	Hypocotyl length	440/665	20–650	24	22	68	7	[35]
	‘Rocket’	Seedlings	Plant height; hypocotyl length	450/670	110	12	18 or 28	76–87 or 56–64	6	[31]
	‘Rocket’	Seedlings	Hypocotyl length	(404, 440, or 455)/660	50	24	23	65	7	[36]
Cabbage (*Brassica oleracea* var. *Capitata*)	Unknown	Seedlings	Hypocotyl length	450/660	50 or 100	24	23	50–55	14	[32]
	Unknown	Seedlings	Hypocotyl length	455/660	100	24	23	65	8	[33]
	Unknown	Seedlings	Hypocotyl length	440/665	100	24 or 16	22	70	8	[30]
	Unknown	Seedlings	Hypocotyl length	455/660	100	24	22	70	8	[34]
	‘Kinshun’	Seedlings	Stem length	470/660	50	16	24	-	30	[37]
Calibrachoa (*Calibrachoa* × *hybrida*)	‘Minifamous Neo Royal Blue’	Cuttings	Shoot length	440/660	40 or 80	16	24	95	21	[19]
	‘Kabloom Deep Blue’	Mature plants	Canopy height; stem length	450/660	50 or 100	24	23	60	15	[43]
	‘Kabloom Deep Blue’	Mature plants	Stem length	455/660	100	24	23	65	72	[42]
	‘Kabloom Deep Blue’	Seedlings and mature plants	Stem length	440/665	100	24 or 16	22	70	25 or 102	[40]
	‘Kabloom Deep Blue’	Seedlings	Hypocotyl length	455/660	100	24	23	65	34	[41]
Cherry tomato (*Solanum lycopersicum* var. *cerasiforme*)	‘Cuty’	Seedlings	Plant height	456/665	205	12	27/18 (day/night)	-	27	[25]
Cucumber (*Cucumis sativus*)	‘Cumlaude’	Seedlings	Hypocotyl length	455/661	100	18	25	65	17	[59]
	‘Cumlaude’	Seedlings	Plant height; hypocotyl length; epicotyl length	455/661	100	18	25	55	17	[27]
	‘Xiamei No.2’	Seedlings	Stem length	454/663	100	16	24/22 (day/night)	60–70	17	[26]
Eggplant (*Solanummelongena*)	‘Kokuyo’	Seedling	Stem height	470/660	20–150	16	24	-	25	[24]
	‘Jingqiejingang’	Seedlings	Plant height	458/661	300	12	28/20 (day/night)	70	35	[23]
Geranium (*Pelargonium × hortorum*)	‘Pinto Premium Salmon’	Mature plants	Canopy height; stem length	450/660	50 or 100	24	23	60	19	[43]
	‘Pinto Premium Salmon’	Mature plants	Canopy height	455/660	100	24	23	65	79	[42]
	‘Pinto Premium Salmon’	Seedlings and mature plants	Stem length	440/665	100	24 or 16	22	70	18 or 101	[40]
Kale (*Brassica napus*)	‘Red Russian’	Seedlings	Hypocotyl length	450/660	50 or100	24	23	50–55	12	[32]
	‘Red Russian’	Seedlings	Hypocotyl length	455/660	100	24	23	65	7	[33]
	‘Red Russian’	Seedlings	Hypocotyl length	440/665	100	24 or 16	22	70	7	[30]
	‘Red Russian’	Seedlings	Hypocotyl length	455/660	100	24	22	70	7	[34]
Marigold (*Tagetes erecta*)	‘Orange Boy’	Mature plants	Plant height	440/650	90	16	25	60	70	[18]
	‘Antigua Orange’	Mature plants	Canopy height	450/660	50 or 100	24	23	60	19	[43]
	‘Antigua Orange’	Mature plants	Stem length	450/660	100	24	23	60	19	[43]
	‘Antigua Orange’	Mature plants	Canopy height	455/660	100	24	23	65	78	[42]
	‘Antigua Orange’	Seedlings and mature plants	Stem length	440/665	100	24	22	70	18 or 74	[40]
Mustard (*Brassica juncea*)	‘Ruby Streaks’	Seedlings	Hypocotyl length	440/665	100	24	22	70	7	[30]
	‘Ruby Streaks’	Seedlings	Hypocotyl length	440/665	250–650	24	22	68	8	[35]
Pea (*Pisum sativum*)	-	Seedlings	Plant height	-	-	8	-	-	60	[39]
Petunia (*Petunia × hybrid*)	‘Baccarat Blue’	Mature plants	Stem length	470/660	70 or 150	12	25		59	[17]
	Dwarf varieties mix	Seedlings	Stem height	-	-	12	25	60–70	79	[21]
	‘Duvet Red’	Mature plants	Canopy height; stem length	450/660	50 or 100	24	23	60	14	[43]
	‘Duvet Red’	Seedlings	Hypocotyl length	455/660	100	24	23	65	35	[41]
	‘Duvet Red’	Mature plants	Stem length	455/660	100	24	23	65	51	[42]
	‘Duvet Red’	Seedlings and mature plants	Stem length	440/665	100	24 or 16	22	70	25 or 102	[40]
	‘Baccarat blue’ and ‘Merlin blue Moon	Mature plants	Plant height	470/660	100	14	25	-	28	[44]
	‘Baccarat blue’	Mature plants	Plant height	450/650	100 or 150	14	25	-	53	[22]
Salvia (*Salvia* *Splendens*)	‘Red Vista’	Mature plants	Plant height	440/650	90	16	25	60	70	[18]
Sesame (*Sesamum indicum*)	‘Gomazou’	Seedlings	Stem length	470/660	80	24	28	-	14	[29]
Sunflower (*Helianthus annuus*)	‘Pacino Gold’ and ‘Pacino Cola’	Mature plants	Stem length	450/650	60	22	18	-	56 or 86	[38]
	‘Teddy Bear’	Mature plants	Stem length; internode length	460/660	60	18	21.5	-	70	[16]
Tomato (*Solanum* *lycopersicum*)	*cry1*	Seedlings	Stem length	447/667	150	18	22/18 (day/night)	70	21	[81]
Tulip (*Tulipa × gesneriana*)	‘lasergame’	Mature plants	Cut flower length; internode length	447/659	200	12	20/10 (day/night)	<60	-	[45]
Watermelon/rootstock (*Citrullus lanatus*/*Cucurbita maxima*)	‘Crimson’/‘Marvel’	Grafted transplants	Scion length	460/660	20–50	16	25/20 (day/night)	98–60	14	[28]

Note: PPFD = photosynthetic photon flux density; RH = relative humidity; if no data but ‘-‘ in the cells, this indicates that the relevant information is unavailable in the literature.

**Table 2:** Plant elongation inhibited by blue light relative to red light from sole-source LED lighting.

Plant Name	Genotype/Variety/Cultivar/Strain	Growth Stage	Elongation Growth Biometrics	Blue/Red LED Peak Wavelength (nm)	PPFD (µmol m^−2^ s^−1^)	Photoperiod (h d^−1^)	Air Temperature (℃)	RH (%)	Treatment Days	Reference
Arabidopsis (*Arabidopsis thaliana*)	col-0, *cry2*, *CRY2-OX*	Mature plants	Hypocotyl length	455/660	100	24	23	65	18	[48]
	*CRY1-OX*	Mature plants	Hypocotyl length	455/660	100	24	23	65	18	[48]
	col-0, ler	Seedlings	Hypocotyl length; plant height	-	120	16	21	70	7 or 30	[77]
	col-0	Mature plants	Hypocotyl length	455/660	100	24	23	65	18	[47]
	*phyAphyBphyCphyDphyE*	Mature plants	Hypocotyl length	455/660	100	24	23	65	18	[47]
Artichokes (*Cynara cardunculus* var. *scolymus*)	‘Green Globe’, ‘Cardoon’, and ‘Violetto’	Seedlings	Plant height	448/666	41 (B)/ 237 (R)	16	22	-	30	[69]
Bamboo (*Phyllostachys edulis*)	‘Moso Bamboo’	Seedlings	Stem length; internode length	450/650	30	-	25	70	14	[73]
Banana (*Musa* spp.)	-	*in vitro* plantlets	Plant height	-	45	16	25	-	30	[82]
Barley (*Hordeum vulgare*)	‘Luch’	Seedlings	Shoot length	451/655	70	16	22–23	-	9	[75]
Bitter Gourd (*Momordica charantia*)	‘QX001’	Seedlings	Plant height	465/650	50	12	25	60–80	-	[62]
Cannabis (*Cannabis* *sativa*)	‘Babbas Erkle Cookies’	Mature plants	Plant height	430/630	250–270/400 (vegetative/flowering stage)	18/12 (vegetative/flowering stage)	28/(19–27) (day/night)	(40–55)/(50–65) (day/night)	70	[76]
Cherry tomato (*Solanum lycopersicum* var. *cerasiforme*)	-	Seedlings	Plant height	450/650	320	12	28/18 (day/night)	60–80	30	[54]
	-	Seedlings	Plant height	-	320	12	28/18 (day/night)	60–80	30	[83]
Chrysanthemum (*Dendranthema grandiflorum*)	‘Cheonsu’	*in vitro* plantlets	Stem length	440/650	50	16	25	70	35	[84]
	‘Token’	Mature plants	Shoot length	469/620	25	-	19	-	119	[64]
Coriander (*Coriandrum sativum*)	‘Sumai’	Seedlings	Plant height	450/660	200	16	24	48	20	[72]
Cucumber (*Cucumis sativus*)	‘Sweet Slice’	Seedlings	Stem length	-	200 or 500	16	25/20 (day/night)	40	16	[52,53]
Cymbidium orchid (*Cymbidium madidum*)	‘Golden Bird’	*in vitro* plantlets	Leaf length	450/660	40	16	25	-	90	[85]
Doritaenopsis orchid (*Orchidaceae*)	-	*in vitro* plantlets	Leaf length	450/660	70	16	25	70	35	[86]
Grape (*Vitis*)	‘Hybrid Franc’, ‘Ryuukyuuganebu’, ‘Kadainou R-1’	*in vitro* plantlets	Plant height; internode length	480/660	50	16	25		30	[87]
	‘Manicurefinger’	*in vitro* plantlets	Stem length	440/630	50	12	25	80	40	[88]
Impatiens (*Impatiens* *walleriana*)	‘SuperElfin XP Red’	Seedlings	Plant height	446/(634 and 664)	160	18	20	-	32 or 33	[57]
	‘SuperElfin XP Red’	Seedlings	Plant height	446/(634 and 664)	160	18	20	-	33 or 34	[58]
Impatiens Balsamina (*Impatiens* *balsamina*)	-	Seedlings	Stem height; hypocotyl length	-	-	12	25	60–70	79	[21]
Kale (*Brassica napus*)	‘Scarlet’	Seedlings	Hypocotyl length	430/660	100	16	24	-	7	[63]
Kiwi (*Actinidia chinensis*)	‘Hayward’	Seedlings	Stem length	470/665	200	16	21	80	21	[71]
Lettuce (*Lactuca sativa*)	‘Okayama-saradana’	Seedlings	Stem height	470/660	20–150	16	24	-	25	[24]
	‘Okayama-saradana’	Seedlings	Stem length	450/660	85 or 170	16	20–22	-	20	[51]
	‘Waldmann’s Green’	Seedlings	Stem length	-	200 or 500	16	25/20 (day/night)	40	21	[52,53]
	‘Green Oak Leaf’	Mature plants	Stem length	460/630	133	14	24/20 (day/night)	60	50	[49]
	‘Rouxai’	Seedlings	Leaf length	449/664	180	20	22.5	44	11	[89]
Maize (*Zea mays*)	‘Zheng58’	Seedlings	Mesocotyl length; coleoptile length	450/660	22 for R; 13 for B	12	22	70	5	[74]
Mamacadela (*Brosimum gaudichaudii*)	-	*in vitro* plantlets	Stem length	-	100	16	25	40	50	[90]
Mint (*Mentha*)	‘Spear mint’, ‘Pepper mint’, and ‘Horse mint’	Mature plants	Plant height	(460–475)/(650–665)	500	16	25	60	60	[91]
Mulberry (*Morus alba*)	‘Longsang No. 1’	Seedlings	Stem length	465/660	100	14	28/23 (day/night)	60–65	20	[70]
Mustard (*Brassica juncea*)	‘Ruby Streaks’	Seedlings	Hypocotyl length	450/660	50	24	23	50–55	11	[32]
	‘Ruby Streaks’	Seedlings	Plant height	450/670	110	12	18 or 28	76–87 or 56–64	8	[31]
Pepper (*Capsicum annuum*)	‘Hangjiao No.12’	Seedlings	Plant height; first internode length	460/660	180	12	24/18 (day/night)	70	30	[61]
	‘HA-2502’	Seedlings	Hypocotyl length; plant height	457/657	300	12	26/18 (day/night)	70	15 or 30	[60]
Radish (*Raphanus sativus*)	‘Cherry Belle’	Seedlings	Stem length	-	200 or 500	16	25/20 (day/night)	40	21	[52,53]
Rehmannia (*Rehmannia glutinosa*)	-	*in vitro* plantlets	Stem length	466/665	100	16	25	40	50	[92]
Rice (*Oryza sativa*)	‘IR1552’ and ‘TS10’	Seedlings	Plant height	460/630	160	12	30/25 (day/night)	70	14	[67]
	‘XZX24’ and ‘HZY261’	Seedlings	Plant height	450/665	100	12	25/15 (day/night)	-	28	[66]
Salvia (*Salvia* *Splendens*)	‘Vista Red’	Seedlings	Plant height	446/(634 and 664)	160	18	20	-	34 or 37	[58]
	‘Vista Red’	Seedlings	Plant height	446/(634 and 664)	160	18	20	-	36	[57]
Soybean (*Glycine max*)	‘Hoyt’	Seedlings	Stem length	-	200 or 500	16	25/20 (day/night)	40	21	[52,53]
	‘Pungwon’	Seedlings	Plant height	447/650	50	24	23	-	5	[93]
Squash (*Cucurbita moschata Duch*.)	‘Strong Man’	Seedlings	Plant height	453/660	150	12	25/20 (day/night)	70	43	[94]
Strawberry (*Fragaria × ananassa*)	‘Akihime’	*in vitro* plantlets	Plant height	450/660	45	16	25	-	30	[95]
Tomato (*Solanum* *lycopersicum*)	‘Early Girl’	Seedlings	Plant height	446/ (634 and 664)	160	18	20	-	31 or 33	[58]
	‘Komeett’	Seedlings	Hypocotyl length	455/661	100	18	25	64.6	21	[59]
	‘Early Girl’	Seedlings	Stem length	-	200 or 500	16	25/20 (day/night)	40	21	[52,53]
	‘Early Girl’	Seedlings	Plant height	446/(634 and 664)	160	18	20	-	31 or 32	[57]
	‘Piennolo’	Seedlings	Plant height; internode length	446/664	190	12	24/18 (day/night)	60–80	16	[55]
	‘Moneymaker’	Seedlings	Stem length	454/663	100	16	24/22 (day/night)	60–70	17	[26]
	‘Moneymaker’	Seedlings	Hypocotyl length; plant height	-	120	16	21	70	7 or 30	[77]
Zinnia (*Zinnia elegans*)	‘Art Deco’	Seedlings	Hypocotyl length; stem height	-	-	12	25	60–70	79	[21]

Note: PPFD = photosynthetic photon flux density; RH = relative humidity; if no data but ‘-‘ in the cells, this indicates that the relevant information is unavailable in the literature.

**Table 3:** Similar plant elongation responses to blue light relative to red light from sole-source LED lighting.

Plant Name	Genotype/Variety/Cultivar/Strain	Growth Stage	Elongation Growth Biometrics	Blue/Red LED Peak Wavelength (nm)	PPFD (µmol m^−2^ s^−1^)	Photoperiod (h d^−1^)	Air Temperature (℃)	RH (%)	Treatment Days	Reference
Arabidopsis (*Arabidopsis thaliana*)	*phot1phot2*	Mature plants	Stem length	455/660	100	24	23	65	20	[46]
	*CRY1-OX*	Mature plants	Stem length	455/660	100	24	23	65	18	[48]
	*phyAphyBphyCphyDphyE*	Mature plants	Stem length	455/660	100	24	23	65	18	[47]
Cabbage (*Brassica oleracea* var. *Capitata*)	‘Red Rookie’	Seedlings	Stem length	470/660	50	16	24	-	30	[37]
Geranium (*Pelargonium × hortorum*)	‘Americana Light Pink Splash’	Mature plants	Stem length; internode length	460/660	60	18	20.7	-	49	[16]
	‘Pinto Premium Salmon’	Seedlings	Hypocotyl length	455/660	100	24	23	65	22	[41]
Kalanchoe (*Kalanchoe blossfeldiana*)	‘Simone’	Mature plants	Shoot length	469/620	25	-	19	-	119	[64]
Kale (*Brassica napus*)	‘Red Russian’	Seedlings	Hypocotyl length	447/660	220	18	21/17 (day/night)	60	-	[79]
Lettuce (*Lactuca sativa*)	‘Cheong Chi Ma’	Seedlings	Shoot length	460/635	200	18	20	60–65	28	[78]
	‘Rouxai’	Seedlings	Leaf length	449/664	180	20	22.5	44	25	[89]
Marigold (*Tagetes erecta*)	‘Antigua Orange’	Mature plants	Stem length	450/660	50	24	23	60	19	[43]
	‘Antigua Orange’	Seedlings	Hypocotyl length	455/660	100	24	23	65	24	[41]
	‘Antigua Orange’	Seedlings	Stem length	440/665	100	16	22	70	18	[40]
Mustard (*Brassica juncea*)	‘Ruby Streaks’	Seedlings	Hypocotyl length	450/660	100	24	23	50–55	11	[32]
	‘Ruby Streaks’	Seedlings	Hypocotyl length	455/660	100	24	23	65	7	[33]
	‘Ruby Streaks’	Seedlings	Hypocotyl length	440/665	100	16	22	70	7	[30]
	‘Ruby Streaks’	Seedlings	Hypocotyl length	455/660	100	24	22	70	7	[34]
	‘Ruby Streaks’	Seedlings	Hypocotyl length	440/665	20–120	24	22	68	8	[35]
	‘Ruby Streaks’	Seedlings	Hypocotyl length	450/670	110	12	18 or 28	76–87 or 56–64	8	[31]
	‘Ruby Streaks’	Seedlings	Hypocotyl length	(404,440, or 455)/660	50	24	23	65	8	[36]
	‘Red Lace’	Seedlings	Hypocotyl length	447/660	220	18	21/17 (day/night)	60	-	[79]
Pepper (*Capsicum annuum*)	‘California Wonder’	Seedlings	Stem length	-	200 or 500	16	25/20 (day/night)	40	21	[52,53]
Petunia (*Petunia × hybrid*)	Dwarf varieties mix	Seedlings	Hypocotyl length	-	-	12	25	60–70	79	[21]
Poinsettia (*Euphorbia pulcherrima*)	‘Novia’	Mature plants	Shoot length	469/620	25	-	19	-	119	[64]
Soybean (*Glycine max*)	‘Pungwon’	Seedlings	Plant height	447/650	50	24	23		0.5–1.5	[93]
Squash (*Cucurbita moschata Duch*.)	‘Strong Man’	Seedlings	Plant height	453/660	150	12	25/20 (day/night)	70	21–30	[94]
Tomato (*Solanum* *lycopersicum*)	‘SV0313TG’	Seedlings	Plant height	457/657	300	12	28/19 (day/night)	70	30	[80]
	‘Moneymaker’, *CRY2-OX3*, and*CRY2-OX8*	Seedlings	Stem length	447/667	150	18	22/18 (day/night)	70	21	[81]
Verbena (*Verbena aubletia*)	-	Seedlings	Stem length; hypocotyl length	-	-	12	25	60–70	79	[21]
Wheat (*Triticum aestivium*)	‘USU-Apogee’	Seedlings	Stem length	-	200 or 500	16	25/20 (day/night)	40	21	[52,53]

Note: PPFD = photosynthetic photon flux density; RH = relative humidity; if no data but ‘-‘ in the cells, this indicates that the relevant information is unavailable in the literature.

## Data Availability

Data are contained within the article.

## Supplementary Materials

The following supporting information can be downloaded at: https://www.mdpi.com/article/10.3390/plants13010115/s1, Table S1: Electrical lighting sources commonly used in controlled-environment plant production and blue lighting sources used in the literature. References [165,166] are cited in the supplementary materials.
