# Supplementary material for: Magic Blue Light: A Versatile Mediator of Plant Elongation
Source: Plants (Basel). 2023 Dec 31;13(1):115. doi: 10.3390/plants13010115 (PMC10780743; doi:10.3390/plants13010115)
Supplement: Supplementary file 1 [file plants-13-00115-s001.zip › plants-2748889-supplementary.pdf]

Supplementary Table S1. Electrical lighting sources commonly used in controlled environment plant production and blue lighting sources used in literature.

Lamp types	BL proportion (%)	B:G:R ratios	Peak wavelength (nm)	Wavelength range (nm)	R:FR ratio	PPS	Brand/Model	Lighting manufacturer	References
Multispectral lighting									
CWF	25						Vita-Lite	Duro-Test Corp., Fairfield, NJ,USA	[10]
CWF	19	19:49:32					F40T12	Philips Lighting, Somerset,NJ, USA	[59]
CWF	18	18:55:27	580	360-800	18	0.86	F96T12/CW/VHO	Osram Sylvania Ltd., Markham, Ontario, Canada	- [160]
CWF			575			0.85	F96T12	Philips, Amsterdam, The Netherlands	[57]
CWF	16	16:70:14	600	350-850				Sylvania	[14]
MH	20	20:56:24	575	300-800	3	0.82	ETAC-400-MH-CH	Energy Technics, York, PA, USA	[12, 99]
MH	23						Optimarc	Tungsten Products Corp., North Bergen, NJ,USA	[10]
HPS	7							GE Lucalox	[10]
HPS	9	9:51:40	569						[165]
HPS			600	425-725		0.86	LU150	Acuity Lithonia Lighting, Conyers, GA	[148]
HPS			600	425-725		0.86	LR48877	P.L. Light Systems, Beamsville, ON, Canada	- [148]
HPS						0.85	NAV T-400W	Osram,Munich, Germany	[145]
RB-LED	10	10:0: 90	453 and 660	425-700		0.88	GP-TOPlight DR/B-LB2013	Philips, Eindhoven, the Netherlands	[148]
RB-LED	12		450 and 650	400-700		0.89	Luxeon Rebel Tri-Star LEDs;	Quadica Developments Inc., Ontario, Canada	[53]
RBG-LED	10	10:5:85	453 and 660	425-700		0.88	GP-TOPlight	Philips, Eindhoven, the Netherlands	[148]

							DR/W-MB2013		
RBG-LED	14	14:23:63	475, 550, and 660	400-700		0.89	Luxeon Rebel Tri-Star LEDs;	Quadica Developments Inc., Ontario, Canada	[53]
Warm white LED	11	11:41:48	450 and 625	400-800		0.84		Multicomp; Newark, Gaffney, SC	[53]
Neutral white LED	19	19:46:35	450 and 600	400-800		0.84		Multicomp; Newark, Gaffney, SC	[53]
Cool white LED	28	28:48:25	440 and 580	400-800		0.83		Multicomp; Newark, Gaffney, SC	[53]
Broad-band blue lighting									
Blue FL			450	350-550			BF6 165-12	JKL Components Corp., Paccoima, CA, USA	[12, 99]
Blue FL			450	400-550	1.87		Philips TLD 18/18	Philips	[11]
Blue FL			435 and 470	400-600			F20 T12/246	Sylvania, Danvers, Mass.	[14]
Blue FL	91		454	396-565	1.28		Philips TL-D 36 W/18	Philips	[166]
Narrow-band blue lighting									
Blue LED			450	400-500		0.49	Royal Blue (LUXEON Rebel	Philips, Amsterdam,Netherlands	[43]
Blue LED			450	425-500		0.5	PHYTOFY® RL LED lighting system	OSRAM GmbH, Munich, Germany	[31]
Blue LED			455	400-500		0.49	LX602C	Heliospectra AB, Gothen-burg, Sweden	[33]
Blue LED			440			0.49	Pro Series 325	LumiGrow, Inc., Emeryville, CA	[30]
Blue LED			405	388-425		0.58	RX30	Heliospectra AB, Gothenburg, Sweden	[36]
Blue LED			440	419-455		0.49	LX601C	Heliospectra AB, Gothen-burg, Sweden	[36]

Blue LED	455	425-482	0.48	Pro650	LumiGrow, Emeryville, USA	[36]
Blue LED	446	400-500	0.48	customized	Osram OptoSemiconductors, Northville, M	[57]
Blue LED	455	400-520	0.51	ISL-305X302 Series,	CCS Inc., Kyoto, Japan	[27]
Blue LED	440			GF-320s	Good Feeling Co. Ltd., Sungnam, Korea	[18]
Blue LED	470	405-530		IS series	CCS Inc., Kyoto, Japan	[29]
Blue LED	470	425-550		Q3J	Phillips LUMILEDS, San Jose, California, USA	[17]
Blue LED	460	400-525	0.58	Luxeon Rebel Tri-Star LEDs;	Quadica Developments Inc., Ontario, Canada	[53]

Note: BL = Blue light; B:G:R ratios = Blue : green : red LED light ratios; R:FR ratio = Red : far-red light ratio; PPS = phytochrome photostationary state; CWF = cool-white fluorescent lamp; HPS = high-pressure sodium lamp; MH = metal halide lamp; Blue FL = Blue fluorescent lamp
